# Use of RNAi With *OsMYB76R* as a Reporter for Candidate Genes Can Efficiently Create and Verify Gametophytic Male Sterility in Rice

**DOI:** 10.3389/fpls.2021.728193

**Published:** 2021-09-06

**Authors:** Yun Chen, Wenping Zhu, Shudan Shi, Lina Wu, Shuanglin Du, Liangshen Jin, Kuan Yang, Wenjia Zhao, Jiaxin Yang, Longbiao Guo, Zhongwei Wang, Yi Zhang

**Affiliations:** ^1^State Key Laboratory for Conservation and Utilization of Bio-Resources in Yunnan, Research Center for Perennial Rice Engineering and Technology in Yunnan, School of Agriculture, Yunnan University, Kunming, China; ^2^College of Agronomy and Biotechnology, Southwest University, Chongqing, China; ^3^State Key Laboratory of Rice Biology, China National Rice Research Institute, Hangzhou, China; ^4^Biotechnology Research Center, Chongqing Academy of Agricultural Sciences, Chongqing, China

**Keywords:** *Oryza sativa*, gametophytic male sterility, *OsMYB76R*, RNAi, GMS preparation and identification

## Abstract

Gametophytic male sterility (GMS) plays an important role in the study of pollen development and seed propagation of recessive nuclear male sterile lines insensitive to the environmental conditions in hybrid rice breeding. Since the inherent phenotypic and genetic characteristics of GMS, it is very difficult to find and identify the GMS mutants. However, due to the abundance of gene transcription data, a large number of pollen-specific genes have been found, and most of them may be associated with GMS. To promote the study of these genes in pollen development and heterosis utilization, in this study, an easy and efficient method of creating and identifying GMS was established using RNAi and *OsMYB76R* as a reporter. First, the *OsC1*/*OsMYB76* gene involved in anthocyanin synthesis was modified, and we have validated that the modified *OsMYB76R* is workable as the same as the pre-modified *OsMYB76* gene. Then, the ascorbic acid oxidase gene *OsPTD1* was downregulated using RNAi, driven by its own promoter that resulted in abnormal pollen tube growth. Finally, the RNAi elements were linked with *OsMYB76R* and transformed into an *osmyb76* mutant, and the distortion of purple color segregation was found in T_1_ and F_1_ generations. This indicates that the *OsPTD1* GMS was prepared successfully. Compared to current methods, there are several advantages to this method. First, time is saved in material preparation, as one generation less needs to be compared than in the conventional method, and mutation screening can be avoided. In addition, for identification, the cost is lower; PCR, electrophoresis, and other processes are not needed; and no expensive chemicals or instruments are required. Finally, the results are more accurate, with much lower background effects, and no damage to the plant. The result is an easy, efficient, low-cost, and accurate method of preparing and identifying GMS genes.

## Introduction

Plant male sterility plays an important role in the study of the development of the pollen mechanism and heterosis utilization. This includes sporophytic male sterility (SMS) and gametophytic male sterility (GMS). These two types are quite different in their heredity and phenotypes. SMS is caused by the sterility of pollen grains, as determined by sporophyte genotypes. In addition, the SMS gene can be transmitted between generations by either female gametes or male gametes in heterozygotes. All pollens in an SMS anther are sterile. In the field, the SMS anthers are usually abnormal in shape, color, and size, and the sterile plant has few seed settings. It is easy to find and identify homozygous SMS mutants. Due to the accessibility of SMS mutants, many studies have been carried out on the mechanism of pollen development and SMS using abundant SMS mutants (Ma, 2005; Wilson and Zhang, 2009; Ariizumi and Toriyama, 2011; Shi et al., 2015). By contrast, GMS is caused by the sterility of the pollen grains, as determined by gametophyte genotypes. In addition, the GMS gene can only be transmitted through the female gamete in heterozygotes, which leads to a very low probability of a homozygous mutant. Half of the pollen grains in a heterozygous anther are fertile, and the other half are sterile. There is no significant difference in appearance between the anthers of mutants and wild types in field observations, and the seed setting radio for the GMS mutant is normal. It is very difficult to locate and identify GMS mutants directly (Han et al., 2006, 2011; Boavida et al., 2009; Chen et al., 2012; Yang et al., 2012). For these reasons, compared with SMS, the location and identification of GMS are more difficult than for SMS. In officially published reports, GMS mutants have usually been created through spontaneous mutation, induced mutation, T-DNA insertion mutation, and so on. After mutation, the GMS candidates were verified in their crossing or self-crossing generations using PCR or resistance to screening agents, or their traits for detecting the segregation distortion of mutated genes or selectable markers or reporters (SariGorla et al., 1996; Feldmann et al., 1997; Howden et al., 1998; Boavida et al., 2009; Yang et al., 2009; Moon et al., 2013; Liu et al., 2016). These strategies, however, require special equipment, expensive chemicals, or invasive treatment, which entails time-consuming, low-efficiency, low-accuracy, high-cost, sample-damaging practices. Further, fewer GMS mutants are produced by these means, and the study of GMS has fallen behind the SMS research.

Pollen development is regulated not only through sporophytic gene expression but also through a large number of pollen genes (Ma, 2005). The genes associated with GMS are usually expressed in microspores and pollen at a late stage of anther development. Due to the development of the transcriptome and other detection methods for gene expression, an increasing number of pollen-specific genes have been identified (Ma, 2005; Suwabe et al., 2008; Wang et al., 2020). It is speculated that most of these genes are related to gametophyte development, which provides us with an opportunity to understand the mechanism of pollen development and GMS. To identify GMS-related genes from all the pollen-specific genes, it is necessary to develop a simple, rapid, economical, and accurate method for the preparation and identification of GMS.

Anthocyanin pigmentation is visible to the naked eye. The rice coleoptile purple line is a clear purple line that appears on each side of the coleoptile after the seed germination. Due to its early performance stage (budding stage) and stability, it is suitable to use as reporter traits (Xi, 1997; Zhang et al., 2004). *OsC1*/*OsMYB76* is a key gene encoding MYB transcription factor for anthocyanin biosynthesis in almost all organs, such as coleoptile in rice (Reddy, 1998; Zhang et al., 2004; Zhao et al., 2016; Sun et al., 2018; Zheng et al., 2019; Hu et al., 2020). Hence, *OsC1*/*OsMYB76* is an ideal reporter for the indication of transgenic events.

In view of the low efficiency and high cost of the preparation and identification of GMS in commonly used methods, this study aimed to design and validate a new method of creating and verifying GMS using RNAi technology and *OsMYB76R* (the revised *OsMYB76*) as a reporter gene. The GMS-related gene *OsPTD1* was identified in rice using our new method. We provide an easy, efficient, and low-cost method of creating and identifying the gene for GMS.

## Materials and Methods

### Plant Materials and Growth Conditions

The *Japonica* rice line Zhonghua 11 was kindly provided by Dr. Li Ping at Shanghai University. The *Indica* line R25, with multiple organs displaying purple color, is a self-breeding restorer, and YR25, without purple color, is an *osmyb76* mutant from R25. Zhongjiu B, another *osmyb76* mutant without purple color in all organs, is a breeding material that features a shorter growth period than Zhonghua 11, and it was kindly provided by the China National Rice Research Institute. Zhongjiu B-*osabcg15* is nuclear male sterility material, an *osabcg15* near-isogenic line of Zhongjiu B, derived from crosses and backcrosses between Zhongjiu B (the recurrent parent) and *osabcg15* (Wu et al., 2014). All rice plants used in this study were grown in the fields in Jinghong, Yunnan, China.

### Vector Construction

*OsMYB76R* was synthesized by Shanghai Generay Biotech Co., Ltd. (Shanghai, China), after eliminating the restriction sites of *OsMYB76*, and its code was optimized according to the rice preference. The target fragment was amplified using the synthesized *OsMYB76R* as a template. The GUS gene in the pCAMBIA1301 vector was substituted with the PCR product of *OsMYB76R* using a homologous recombination strategy described in the user manual of the In-Fusion HD Cloning Kit (Takara, Otsu, Japan), which generated the *pP35S-OsMYB76R*-OE vector. This vector was transformed into the Zhongjiu B calli using an agrobacterium-mediated method. The sequences of *OsMYB76R* and primers 76F/R are shown in the Supplementary Material 3.

For the RNAi of the *OsPTD1* gene, all of the RNAi elements, such as the promoter of *POsPTD1* or *P35S*, target sequence (left arm), stem-loop structure (first intron of *OsMYB76*), complementary sequence of target (right arm), and Tnos, were synthesized as one fusion sequence by Shanghai Generay Biotech Co., Ltd. The whole RNAi sequence was loaded between the restriction sites *Sal*I and *Sbf*I of pCAMBIA1301, which generated the vectors *pPOsPTD1-OsPTD1*-RNAi and *pP35S-OsPTD1-RNAi_P35S-OsC1*. These two vectors were separately transformed into Zhonghua 11.

For the preparation and identification of *OsPTD1* GMS, the synthesized RNAi sequences were inserted into *pP35S-OsMYB76R*-OE using *Sal*I and *Sbf*I, which generated *pPOsPTD1-OsPTD1_P35S-OsMYB76R* and *pP35S-OsPTD1_P35S-OsMYB76R*. These were transformed into Zhongjiu B.

### Pollen Germination *in vitro*

The germination medium was composed of 18% sucrose, 5% potato starch, and 0.005% H_3_BO_3_. The solid medium was prepared in advance following these steps: evenly mixing the ingredients, heating and melting them in a microwave oven, cooling, and evenly spreading them on the slide. Immediately after the floret opened, pollen grains were shed onto the solid germination medium. After 30 min incubation at 28–30°C, the slides were observed with a Nikon (Tokyo, Japan) SMZ1500 stereoscope and photographed with a Nikon DS-5Mc digital camera.

### Coleoptile Purple Color Observation and Inheritance Analyses

The transgenic positive plants with anthocyanin color were self-crossed to obtain the T_1_ generations. The same positive plants were used as male parents to pollinate the male sterile of Zhongjiu B-*osabcg15* to obtain F_1_ generations. After being soaked and pregerminated, the seeds were evenly planted in a mud tray, covered with plastic cling to keep them wet, and placed under natural light. The purple color of the coleoptile was investigated after 2–3 days of growth. The members of the segregated groups purple and no-purple were counted and calculated, and a chi-square test was conducted to establish whether the observed values were consistent with the theoretical values.

## Results

### *OsMYB76R*, Revised From *OsC1*/*OsMYB76*, Is Workable When Replacing the *GUS* Gene as a Reporter in Rice

**FIGURE 1:**
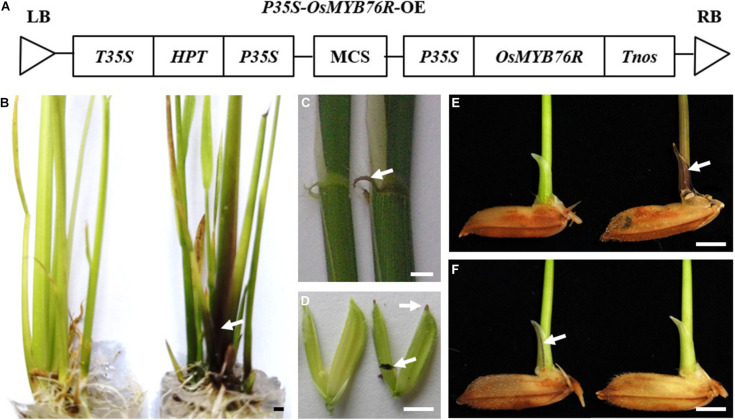
Phenotype of the *OsMYB76R* gene. **(A)** The structure of the *pP35S*-*OsMYB76R*-OE vector. The left sides of panels **(B–E)** refer to the color of different organs in Zhongjiu B, and the right sides refer to the color of different organs in a plant that is positive for *OsMYB76R* overexpression. The traits in panels **(B–D)** are from T_0_ plants. The trait in panel **(E)** is from the T_1_ plant. The left side of the panel **(F)** is purple wild type R25, and the right side of the panel **(F)** shows *osmyb76* mutant YR25. The arrows point to the purple tissue. Scale bars, 0.2 cm.

The *OsC1*/*OsMYB76* gene is a functional gene for anthocyanin biosynthesis in rice. In the previous studies, the CDS (coding sequence) of the *OsC1*/*OsMYB76* gene was used to replace the *GUS* gene in the vector pCAMBIA1301. The function of the *OsC1*/*OsMYB76* gene was verified with a complement test (data not shown). Transgenic positive plants showed a purple color in many organs, such as the stem base of the seedling, the leaf sheath, the auricle, the stigma apiculus, and the coleoptile, which shows that it can be used as a reporter gene to indicate positive transgenic events. The CDS of *OsMYB76* contains common restriction sites, such as *Pst*I, *Sac*I, and *Sal*I, but if *OsMYB76* is directly used as a reporter, it must influence the freedom of restriction site selection when the target gene is loaded. To remove these restriction sites, we optimized and modified the gene with artificial synthesis and obtained *OsMYB76R*, after that we replaced the *GUS* gene in pCAMBIA1301 with homologous recombination, which resulted in the functional verification vector *pP35S-OsMYB76R*-OE (Figure 1A). The plasmid was transformed into Zhongjiu B without any purple in any organs, and the results showed that the stem base of the seedling, the leaf sheath, the auricle, the stigma and apiculus, and the coleoptile of the transgenic positive plants were all purple in color (Figures 1B–E). The distribution of purple coloration of the coleoptile of the transgenic-positive plants was wider and easier to observe than that of most wild-type lines, such as R25 (an *Indica* restorer line) (Figures 1E,F). The results showed that the revised *OsMYB76R* gene has the equivalent of a genetic function for *OsMYB76* in rice anthocyanin biosynthesis, and *OsMYB76R* is suitable for reporting transformants.

### RNA Interference of *OsPTD1* Damages Pollen Tube Development

**FIGURE 2:**
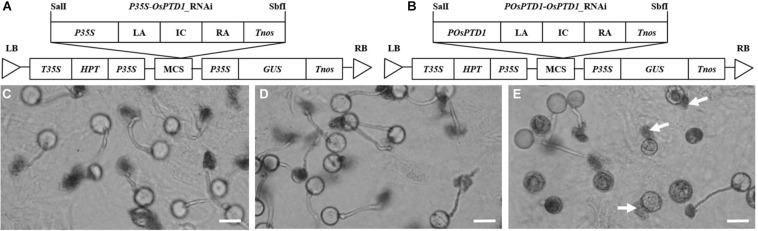
RNAi pollen germination *in vitro.* **(A)** The structure of *pP35S*-*OsPTD1*-RNAi. **(B)** The structure of *pPOsPTD1*-*OsPTD1*-RNAi. **(C)** The germination of wild pollen. **(D)** The germination of the *P35S*-promoted RNAi pollen. **(E)** The germination of *POsPTD1-*promoted RNAi pollen; arrows indicate the rupture of RNAi pollen. All images were created 30 min after germination. Scale bars, 20 μm.

The ascorbate acid oxidase (AAO) gene *NPT303* plays an important role in the growth of the tobacco pollen tube, and the silencing of *NPT303* results in the abnormal growth of the pollen tube (de Groot et al., 2004). There are more than 10 AAO genes in the rice genome,^1^ and most of them have an unclear function. Among these AAO genes, *Os05g0485800* encodes a protein that is highly homologous to NPT303, may be related to pollen tube growth, and is named pollen tube development gene 1 (*OsPTD1*). To determine whether it plays a role in pollen germination and growth, two vectors for *OsPTD1* RNA interference were constructed, promoted by the constitutive 35S CaMV promoter (*P35S*) and the specific promoter of *POsPTD1* (Figures 2A,B), and were transferred into Zhonghua 11. The T_0_ pollen germination assay showed that all the pollen from the RNAi promoted by *P35S* and the wild type could be germinated and grown normally, but only about half of the pollen from the RNAi promoted by *POsPTD1* germinated and grew normally. The germination of other pollen grains was abnormal, with most of the abnormally germinated pollen tubes rupturing without growing (Figures 2C–E). These results show that the RNAi under the *OsPTD1* promoter broke the growth of the pollen tube, and this may result in GMS.

### Construction and Verification of a System for GMS Preparation and Identification

**FIGURE 3:**
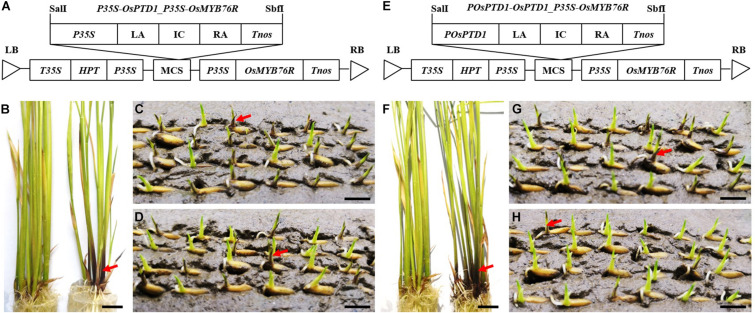
Inheritance characteristics of *OsPTD1* RNAi. Panels **(A,E)** depict the structure of the RNAi vectors *pP35S-OsPTD1*_*P35S*-*OsMYB76R* and *pPOsPTD1*-*OsPTD1*_*P35S*-*OsMYB76R*, respectively. Panels **(B–D)** show a T_0_-positive plant, and a T_1_ and F_1_ (positive plants as the male parents) of *P35S-OsPTD1*_*P35S-OsMYB76R*. Panels **(F–H)** show the T_0_-positive plants and T_1_ and F_1_ (positive plants as the male parent) of *POsPTD1-OsPTD1*_*P35S-OsMYB76R*. Scale bars, 0.8 cm.

To confirm *OsPTD1* as a gene related to GMS, two vectors for the preparation and identification of GMS were designed and constructed, based on the *OsPTD1* gene. The *P35S-* and *POsPTD1-*promoted *OsPTD1* RNAi elements were linked with *OsMYB76R* that generated the RNAi vectors of *P35S-OsPTD1*_*P35S-OsMYB76R* and *POsPTD1-OsPTD1*_*P35S-OsMYB76R*, respectively (Figures 3A,E). In addition, they were separately transferred into Zhongjiu B (Figures 3B,F). Two types of RNAi-positive plants (purple) were crossed as male parents with a male-sterile Zhongjiu B-*osabcg15* mutant with no purple color. The male parents were also allowed to self-cross. Purple segregations in the coleoptile of F_1_ and T_1_ were investigated 3 days after the seed germination in wet soil in a disk (Figures 3C,D,G,H). The results showed that the T_1_ and F_1_ of *P35S* promoted RNAi exhibiting 3:1 and 1:1 segregation (purple: no purple), respectively, which did not match with the genetic characteristics of GMS. However, the color segregation ratio of the T_1_ of the *POsPTD1* promoted RNAi that did not show a 3:1 ratio but a 1:1 ratio (Table 1). Only a few seeds, namely, F_1_ produced by *POsPTD1-*promoted RNAi male plants, displayed a purple coleoptile, which indicated a distorted segregation ratio, compared with the expected 1:1 (Table 1). The distorted segregation ratio of the *POsPTD1-*promoted RNAi generation is just the genetic characteristics of GMS, which indicates that *POsPTD1-*driven RNAi produced a partial gametophytic defect. The above results indicate that the preparation of *OsPTD1-*related GMS materials using the newly designed method was successful. Hence, this method can identify GMS easily, rapidly, and economically, 3 days after the seed germination.

**TABLE 1:** The segregation ratio of coleoptile purple color in generations of *OsPTD1* RNAi.

Parents or combinations	Generation	Purple color: purple colorless		
		
		Obs ratio	Exp ratio	χ^2^_0.05,1_
*P35S-OsPTD1*RNAi_*P35S-OsMYB76R-1*(A)	T_1_	143:57	3:1	1.13
*P35S-OsPTD1*RNAi_*P35S-OsMYB76R-2*(B)	T_1_	152:48	3:1	0.06
Zhongjiu B-*osabcg15* × A	F_1_	98:87	1:1	0.54
Zhongjiu B-*osabcg15* × B	F_1_	82:72	1:1	0.53
*POsPTD1-OsPTD1*RNAi_*P35S-OsMYB76R-1*(C)	T_1_	112:88	1:1	2.65
*POsPTD1-OsPTD1*RNAi_*P35S-OsMYB76R-2*(D)	T_1_	103:88	1:1	1.03
Zhongjiu B-*osabcg15* × C	F_1_	29:227	0:256	–
Zhongjiu B-*osabcg15* × D	F_1_	18:170	0:188	–

## Discussion

### *OsMYB76R* Is a Favorable Reporter Gene

It is best to identify the positive transformants as soon as possible after transformation. The reporter gene is a visible indicator of whether the target gene is transferred or not, and it can also be widely used for identifying positive transgenic plants. An ideal reporting gene should have the following characteristics: stable phenotype, easy detection, less background affect, low cost, and no damage to plants, the environment, or health (Ziemienowicz, 2001; Rosellini, 2012; He et al., 2020).

The endogenous genes of some plants have been screened and used as reporter genes. For example, anthocyanin-related genes are among the most widely used endogenous reporters in plants, such as maize, wheat, *Arabidopsis*, tobacco, and tomato (Rosellini, 2012; He et al., 2020). The anthocyanin biosynthesis pathway involves many genes, and at least 16 are involved in the anthocyanin biosynthesis in rice (Sharma and Dixon, 2005; Oshima et al., 2019). Due to the limited carrying capacity of the vector, it is impossible to load all genes controlling the anthocyanin synthesis into the same vector as the reporter genes. Scientists often use a compromise strategy to solve this problem: selecting the appropriate genetic background material, one in which most anthocyanin synthesis genes are functional as the transgenic host, and using one to several anthocyanin genes as the reporter (Lloyd et al., 1992; Goldsbrough et al., 1996; Rosellini, 2012; Oshima et al., 2019).

*OsC1*/*OsMYB76* is a homologous gene of the anthocyanin biosynthesis gene *C1* in maize, and it is a functional gene for anthocyanin biosynthesis in rice. The purple color phenotype controlled by *OsC1*/*OsMYB76* is visible and stable, which indicates that it is suitable for use as a reporter gene (Xi, 1997; Reddy, 1998; Zhang et al., 2004; Zheng et al., 2019; Hu et al., 2020). To make it easier to use this gene, *OsMYB76R* was optimized from *OsMYB76* and the usual restriction sites in *OsMYB76* CDS were removed. The revised *OsMYB76R* had normal functions, such as those of *OsMYB76*, and could indicate positive transformants quickly, directly, accurately, and harmlessly in the early T_0_ generation. Additionally, it was easy to separate the positive plants from the next generations of transgenic plants. The *OsMYB76R* sequence was only 819 bp that saved space for the loading of more and longer target genes. Moreover, the usual restriction sites in *OsMYB76R* were removed, which did not affect the freedom of restriction-site selection, when the target gene is loaded. For this reason, *OsMYB76R* is a desirable reporter gene.

In addition, Zhongjiu B is an *Indica-type* rice with a shorter growth period than Zhonghua 11 (a commonly used *Japonica* variety in rice transgene). The transgenic efficiency of Zhongjiu B is higher than the two popular *Indica* rice types, Minghui 63 and 9311. Zhongjiu B, transformed by only one reporter gene *OsMYB76R*, displays purple color in multiple organs, which indicates that other anthocyanin pigment genes in Zhongjiu B meet genetic conditions sufficient to anthocyanin expression in multiple organs. Therefore, Zhongjiu B is a good *Indica* rice host for the transformation of the *OsMYB76R* gene as a reporter.

### Combining Candidate Gene RNAi and *OsMYB76R* to Enable High-Efficiency, Accurate, and Low-Cost Preparation and Identification of GMS

Gametophytic male sterility mutants have traditionally been obtained through natural mutation, induced mutation, T-DNA insertion mutation, and gene editing. After mutagenesis, it is necessary to determine whether the plants are GMSs according to the genetic characteristics of their offspring. This can be done with genotype analyses through PCR or chemical resistance analyses using screening-marker genes in self-crossing generation and the F_1_ generation (mutant as the male parent). Thereafter, segregation distortion is used to determine whether the mutant is GMS. In other words, if the segregation ratio of the self-bred offspring is 1:1 instead of 3:1, and the mutated alleles do not transfer through pollen learned from F_1_, it is GMS (Feldmann et al., 1997; Howden et al., 1998; Boavida et al., 2009). For example, *TMS1* (Yang et al., 2009), *OsGT1* (Moon et al., 2013), and *gaMS-1* (SariGorla et al., 1996) were prepared and determined as GMS using the above methods. These methods are cumbersome and involve the use of multiple reagents and equipment, which inevitably leads to the problems of low efficiency, high cost, low accuracy, and sample damage. For the gene-editing strategy, scientists must spend another generation to remove the editing component to overcome continuous editing and screen out mutants with no transgenic elements that undoubtedly lengthen the identification time by at least one generation.

In this study, using a strategy that combines RNAi technology and *OsMYB76R* reporting technology, a method was designed that allows GMS preparation and identification at the same time by transferring the linked *OsPTD1*-RNAi element and *OsMYB76R* into Zhongjiu B with no purple color in any organ, the GMS genetic resources of *OsPTD1* were prepared successfully. The genetic identification of GMS was completed only 3 days after the seed germination, using purple segregation distortion in the T_1_ and F_1_ progenies, derived from T_0_ positive plants of RNAi.

There are three strategies used to create GMS: random mutations (such as spontaneous mutation, induced mutation, and T-DNA insertion mutation), gene editing (ZFNs, TALENs, CRISPR-Cas, and so forth), and downregulation of gene expression (with RNAi and anti-RNA). The third strategy was used in this study. RNAi is a much more efficient, easier, and cheaper means of GMS preparation than the random mutation strategy, because the burdensome mutant screening work can be avoided in RNAi. This step is necessary for random mutation because there is no definite target gene. Furthermore, the RNAi strategy is quicker than gene editing, where editing elements must be removed in generation T_1_ to avoid any influence on the flowing study by persistent editing. So, if a gene-editing strategy is used to produce GMS, the genetic population for segregation distortion analysis could only be constructed in T_1_, not in T_0_, while it could be finished in T_0_ when the RNAi strategy is adopted. This means that the time required for one generation will be saved. In identification, the first two strategies require PCR, resistance detection, or fluorescence testing to analyze the inheritance behavior, which entails the use of specialized equipment and expensive chemicals and invasive sampling procedures. However, in the RNAi-linked *OsMYB76R* strategy, we use anthocyanin-related genes as reporter genes to analyze their genetic characteristics, in a more direct, simple, timeless, harmless, low-cost, and high-accuracy way than commonly used methods based on PCR detection, resistance testing, or widely used reporting genes, such as *GUS*, *FPs*, and *Luc*.

**FIGURE 4:**
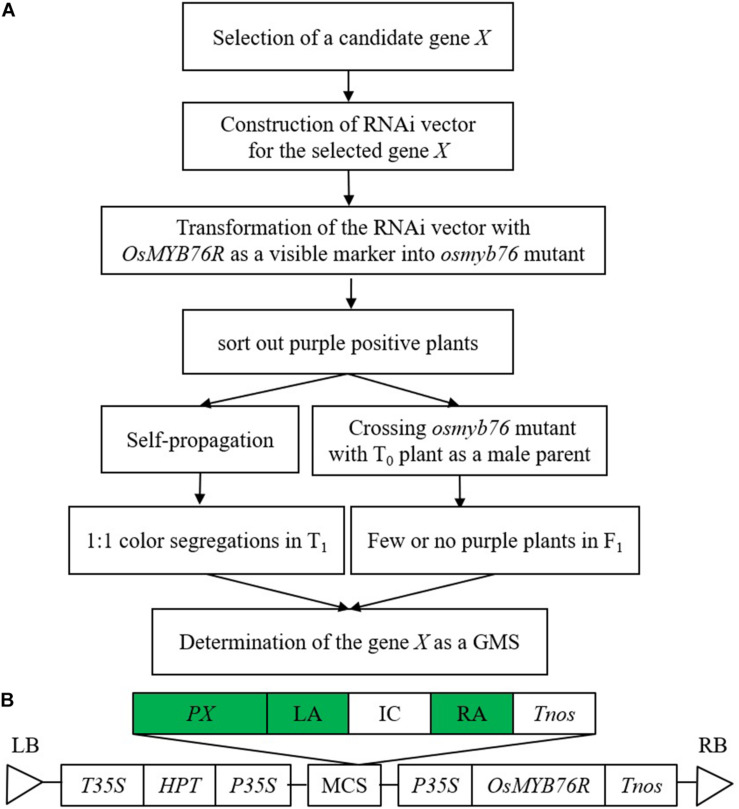
Strategy for gametophytic male sterility preparation and identification. **(A)** Schematic diagram of gametophytic male sterility (GMS) preparation and identification. **(B)** Vector structure of this strategy. When this strategy is used, the green highlighting should be replaced according to the candidate GMS-related gene. X, GMS candidate; P*X*, promoter of *X*; LA, left arm of RNAi-*X*; IC, the first intron of *OsC1*; RA, right arm of RNAi-*X*.

In conclusion, our strategy for the preparation and identification of GMS has the advantages of being harmless, quick, efficient, cheap, accurate, and reliable. In addition, pollen lethal factor and fluorescent protein are the two key components in the present technology of nuclear sterility seed production. If GMS produced by this method is entirely aborted (no transgenic element transfer through pollen), the RNAi elements and linked *OsMYB76R* can be used to replace the lethal pollen factor and the fluorescent protein gene in the current system that is useful for developing a new nuclear-sterility seed-preparation system. Obviously, this strategy can also be used to prepare and identify GMS in other crops. A simple sketch map for understanding this method is shown in Figure 4.

## Data Availability Statement

The original contributions presented in the study are included in the article/Supplementary Material, further inquiries can be directed to the corresponding authors.

## Author Contributions

YC, WZ, and SS designed and performed the experiments. LJ, KY, WZ, and JY performed the experiments and analyzed the data. LW and SD designed the experiments. LG, ZW, and YZ designed the experiments and wrote the manuscript. All authors read and approved the final manuscript.

## Conflict of Interest

The authors declare that the research was conducted in the absence of any commercial or financial relationships that could be construed as a potential conflict of interest.

## Publisher’s Note

All claims expressed in this article are solely those of the authors and do not necessarily represent those of their affiliated organizations, or those of the publisher, the editors and the reviewers. Any product that may be evaluated in this article, or claim that may be made by its manufacturer, is not guaranteed or endorsed by the publisher.

## Footnotes

1
http://www.gramene.org/

## Supplementary Material

Supplementary Material 1 Sequence of synthesized *OsMYB76R.*

Supplementary Material 2 Sequence of synthesized *OsPTD1* RNAi elements.

Supplementary Material 3 Primer sequences for *OsMYB76R* amplification.

## Abbreviations

GMS: gametophytic male sterility
SMS: sporophytic male sterility
MYB: myeloblastosis
AAO: ascorbic acid oxidase.
