# Supplementary material for: Use of RNAi With OsMYB76R as a Reporter for Candidate Genes Can Efficiently Create and Verify Gametophytic Male Sterility in Rice
Source: Front Plant Sci. 2021 Sep 6;12:728193. doi: 10.3389/fpls.2021.728193 (PMC8451479; doi:10.3389/fpls.2021.728193)
Supplement: Supplementary Material 1 — Sequence of synthesized OsMYB76R. [file Data_Sheet_1.doc]

Supplementary data for

Use of RNAi with *OsMYB76R* as a reporter for candidate genes can efficiently create and verify gametophytic male sterility in rice

Yun Chen^1, †^, Wenping Zhu^1, †^, Shudan Shi^1, †^, Lina Wu^2^, Shuanglin Du^1^, Liangshen Jin^1^, Kuan Yang^1^, Wenjia Zhao^1^, Jiaxin Yang^1^, Longbiao Guo^3, 🖂^, Zhongwei Wang^4, 🖂^, Yi Zhang^1, 🖂^

^1^ State Key Laboratory for Conservation and Utilization of Bio-resources in Yunnan, Research Center for Perennial Rice Engineering and Technology in Yunnan, School of Agriculture, Yunnan University, Kunming 650091, Yunnan, China

^2^ Southwest University, Chongqing 401329, China

^3^ State Key Laboratory of Rice Biology, China National Rice Research Institute, Zhejiang 310006, China

^4^ Biotechnology Research Center, Chongqing Academy of Agricultural Sciences, Chongqing 401329, China

^†^ These authors contributed equally to this study

^🖂^ Longbiao Guo, e-mail: guolongbiao@caas.cn

^🖂^Zhongwei Wang, e-mail: shengran4@126.com

^🖂^Yi Zhang, e-mail: zhangyi6116@ynu.edu.cn

Supplementary Material 1 | Sequence of *OsMYB76R*

acgggggactcttgaccatgatgggacgcagggcttgctgtgcaaaggaaggaatgaagcggggtgcctggacgtccaaggaggacgatgtcctggcgagctacattaagtctcacggagagggtaagtggcgggaggttccgcagagagccggacttcggagatgcggcaagtcctgtcgccttagatggttgaactatctccgcccaaatatcaagaggggcaacattgacgatgacgaggaagagctgatcgtgcggcttcatacgctcctggggaatagatggtccctgatcgcaggccgccttccgggaaggaccgataacgagattaagaactactggaatagtacactctccaggaagatcggaacggccgcgaccgccgccgccggctcacgcggcggctccacccctgatacagccagggcgactgacgcggcttccagctcttcagtggtcccaccaggacagcaacagcaaccagcaagccgggcggatactgacaccgcaaccgccgccgccgccgccgccgccactacaacgactgtttgggctccaaaggcagtgcggtgcacgagaggcttctttttccacgatagagaaactgcaccattggctgctgcggcgcctgctcccgcaggagagctgggagacggagatgacgtggattgcgactactattgttctggttcgagttccgctgcaaccacaacctcctcctcctccctcccggttgtggtcgagccatgctttagtgccggcgatgactggatggatgacgtccgcgccttggcgtcattcctcgacacagatgacgcttggaacctgtgtgcgtgagtgaccagctcgaatttccc

Highlights with yellow color refers to the backbone sequences of pCAMBIA1301 which was used as homologous recombination arm. The rest sequence is for the ORF of *OsMYB76R*.

Supplementary Material 2 | Sequence of *OsPTD1* RNAi elements

gtcgacgtcccatgtcaccgacagtactaaatgggtaaagattggataaagtatatggggtatttgtgaggtattattagaaaacttcgtgtggttttgatggacctgttttatgtgttgaaaatatgaatggttatagggtgtgtttgcaagtgcaggatgggaactcatccctcctgcacgcaaaacggagcggctttttaacacatgattaattaaatattagctaatttttttaaaaaaaatggattaatttgatttttttaagcaactttcatatagaaattttttgcaaaaaacacaccgtttaatagtttaaaaacgtgcgcgcgaaaaacgagggagaggggttgggaacatgggtttgcaaacacaaccatagtattggcgattccttttcgtttgagtaaattttacaaaactacaggtattttgaccaaattatcacaaaactacagatttaaggagttgtatcataaaactacacatttagcatcaaatttatcacaaaactgcagattttaggttaagtatcacaaaaatacatatttaatattgaacttatcacaaaactataacttttggagtttaaatccctagcaccattgttatggtggagctataaacattattactttgtgattaaattggttctaaacctttagttttatgataatttagtaactaaacgtgtagttttgtaacacttcatctttaatatgtagttttgtgctaaatttggtgctaaatgtgtaattttgtgatataattccttaaatatgtagttttgtgatagtttggttataatatctgtagttttatgaaatttactcttttcgttttcactgcaatttggaatgatggaattgactagatccggcattaccgatgggctgccgaacgctgtgatgcggttgatcttgagcgatccgggacgccacaagcaccgatgggttctgggagttcatacggctggtgcagcagtgtgtcaatagcagccgggatgtgcgcccaaccatggtcgccgtcgagaggaggatcgaagacatcctgaactcggttgtcaggtcatccaccaccgggttcatgactgccggaggcgacacacccagcaacgagccaaatcgtgaagataacggaaacgagccaaatcccagcaacgagatcgccagggactagtagtacgtacagcagtggtgatttgtcatataggtgtatatcggctgttttcgcatctcaaggcctcaagcagtgtgtgcaatctggagtagtatataaatatgtaaaatgttcatttcgatatactgtcaaatgcgtgtaaattaaccaatgctaaaacaacacactgtgactaaatttactgagttggatgatgaggatgattatgttgcgtgcacacctgatcaggaggacatataatataggccatttgggccgtcttggacaccaccgtttgatttgtatgaagttgggccgaactatgcaagcccagaggcgctgcctctgtgccacggcccacgggcatcgctggatggtcaagcaggtgatcggtggagcgccaatggcggcggcgagacacacagcgcggcgcgcgcgcgaacgtgcggacgcgcgcgccccggccacggccgccgcgctcgtctcctggcctcccgcgcccgctacaaatggcggccccggcgtcccctcctcactccgaagcttcccggttgacgacctctccggtctcccccctcaccccaccgcaacccgggacgtcttccatggccgccgccgccgccgcccccgcctactaaaccaccctacccaccccctccaaactcccacacattacatccttcaaagagagcatcacacacacacacacaccagcctagcgatcacatttccacggaattcgcagaaggtgatcctgatcaacggcgagttcccgggcccgcggatcaactgctcgtccaacaacaacatcgtggtgaacgtgttcaaccagctggacgagccgctgctcttcacctggaacgggatgcagcaccgcaagaactcgtggcaggacggcctcgccgggacgcagtgccccatcgcgccgggcaccaactacacgtacaagtggcagcccaaggaccagatcggcagcttcttctacttcccgtcgctggggatgcaccgcgccgccggcggctacggcgggatcagcgtcgtcagccgcctgcagatctacgagctggtgagctagctattacctaatcgatcgatggtcatcgatcatgagatgatgatgatgagatttgtacttaattgtgatctgtatggatgctgttgttgatcaagttcttgcgatcgatcgatctgaattttcaggtttgaggggtgaccgcaggcggctgacgacgctgatcccgccgtagccgccggcggcgcggtgcatccccagcgacgggaagtagaagaagctgccgatctggtccttgggctgccacttgtacgtgtagttggtgcccggcgcgatggggcactgcgtcccggcgaggccgtcctgccacgagttcttgcggtgctgcatcccgttccaggtgaagagcagcggctcgtccagctggttgaacacgttcaccacgatgttgttgttggacgagcagttgatccgcgggcccgggaactcgccgttgatcaggatcaccttctgcggcgcgccgaatttccccgatcgttcaaacatttggcaataaagtttcttaagattgaatcctgttgccggtcttgcgatgattatcatataatttctgttgaattacgttaagcatgtaataattaacatgtaatgcatgacgttatttatgagatgggtttttatgattagagtcccgcaattatacatttaatacgcgatagaaaacaaaatatagcgcgcaaactaggataaattatcgcgcgcggtgtcatctatgttactagatcgggcctgcagg

Highlights with yellow color refers to restriction sites, the promoter of *OsPTD1* is marked with green color, the RNAi target sequences are highlighted with red color, the first intron of *OsMYB76* is marked with blue color, and Tnos is marked with gray color.

Supplementary Material 3 | Primer sequences for OsMYB76R amplification.

76F: acgggggactcttgaccatg

76R: gggaaattcgagctggtcac
